# Auditory map reorganization and pitch discrimination in adult rats chronically exposed to low-level ambient noise

**DOI:** 10.3389/fnsys.2012.00065

**Published:** 2012-09-11

**Authors:** Weimin Zheng

**Affiliations:** The Neurosciences InstituteSan Diego, CA, USA

**Keywords:** experience-dependent plasticity, neural plasticity, tonotopic map, auditory operant task, auditory cortex, pitch discrimination, adaptation, rats

## Abstract

Behavioral adaption to a changing environment is critical for an animal's survival. How well the brain can modify its functional properties based on experience essentially defines the limits of behavioral adaptation. In adult animals the extent to which experience shapes brain function has not been fully explored. Moreover, the perceptual consequences of experience-induced changes in the brains of adults remain unknown. Here we show that the tonotopic map in the primary auditory cortex of adult rats living with low-level ambient noise underwent a dramatic reorganization. Behaviorally, chronic noise-exposure impaired fine, but not coarse pitch discrimination. When tested in a noisy environment, the noise-exposed rats performed as well as in a quiet environment whereas the control rats performed poorly. This suggests that noise-exposed animals had adapted to living in a noisy environment. Behavioral pattern analyses revealed that stress or distraction engendered by the noisy background could not account for the poor performance of the control rats in a noisy environment. A reorganized auditory map may therefore have served as the neural substrate for the consistent performance of the noise-exposed rats in a noisy environment.

## Introduction

Experience-dependent plasticity of the brain is critical for behavioral adaptation to changing environments. Such plasticity is most dramatic during the critical period in juveniles (Moucha and Kilgard, 2006; Dahmen and King, 2007; Keuroghlian and Knudsen, 2007; Eggermont, 2008; Barnes and Finnerty, 2010). In the auditory system, chronic low-level noise exposure has been shown to be detrimental to the development of the central auditory system (Zhang et al., 2001, 2002; Chang and Merzenich, 2003; Aizawa and Eggermont, 2007; Speechley et al., 2007; Xu et al., 2010). In young rats and cats, continuous exposure to white noise prevents development of a tonotopic map and refinement of neural response selectivity in the primary auditory cortex (A1) (Zhang et al., 2001; Chang and Merzenich, 2003; Aizawa and Eggermont, 2007; Speechley et al., 2007). Rearing in a continuous-noise environment affects vocal learning in songbirds (Marler et al., 1973; Iyengar and Bottjer, 2002) and disrupts development of the auditory space map in barn owls (Efrati and Gutfreund, 2011). Early abnormal sound experience in rat (Han et al., 2007) and mice pups (Takahashi et al., 2006) modified the tonotopic map in A1 and impaired frequency discrimination in adult rats (Han et al., 2007).

The extent to which experience shapes brain function in adult animals is generally believed to be much more limited and is largely contingent on behavioral context, i.e., attention, reward, or extensive training (Keuroghlian and Knudsen, 2007). Recent studies in adult cats exposed to moderate-level tone pips have, however, shown reorganization of the tonotopic map in A1 (Pienkowski and Eggermont, 2009, 2010b,c), demonstrating a marked capacity for experience-dependent plasticity of the brain even in adult cats. Such dramatic influence of sound exposure on the auditory map has, however, not been examined in other species. To date, no study has examined the behavioral effects of low-level noise exposure on adult animals.

We found that after 30 days of exposure to low-level noise, neural networks in the A1 underwent a large-scale reorganization, resulting in transformation from a topographic to a spatially patched/clustered representation of sound frequencies. Consistent with recent studies in adult cats (Pienkowski and Eggermont, 2009, 2010a,c), these results demonstrate a similar degree of environmental influence on the brain representation of sensory information in adult rats as that reported in juveniles of rats (Moucha and Kilgard, 2006; Dahmen and King, 2007; Keuroghlian and Knudsen, 2007; Eggermont, 2008; Barnes and Finnerty, 2010) and other species (Marler et al., 1973; Iyengar and Bottjer, 2002; Aizawa and Eggermont, 2007; Speechley et al., 2007; Eggermont, 2008; Efrati and Gutfreund, 2011; for reviews see: Moucha and Kilgard, 2006; Dahmen and King, 2007; Keuroghlian and Knudsen, 2007; Barnes and Finnerty, 2010). Behaviorally, the noise-exposed adult rats showed impairments in detecting fine pitch variations embedded in a tone sequence. Therefore, living with noise leads to a dramatic reorganization of the auditory map in the A1 and a concomitant impairment in fine but not coarse pitch discrimination. When tested in a noisy environment, the noise-exposed rats performed as well as in a quiet room whereas the control rats performed significantly worse than in a quiet room. Analyses of behavioral patterns of the control rats in a noisy environment indicated that stress or distraction, potentially engendered by the noisy background did not likely contribute to their poor performance. Therefore, the reorganized auditory map in A1—and perhaps, plastic changes in the whole central auditory system (see “Discussion”)—might constitute the neural substrate that underlies the consistent performance of the noise-exposed rats in a noisy environment.

## Materials and methods

The experimental procedures used in this study were approved by the Institutional Animal Care and Use Committee of the Neurosciences Institute and were performed in strict accordance with the US Public Health Service (PHS) Policy for Humane Care and Use of Laboratory Animals (PHS Animal Welfare Assurance no. A4558-01).

### Acoustic experience

50 days old male rats (Sprague-Dawley, Harlan) were ordered from Harlan. Upon arrival, they were placed in individual wire cages in a sound room (Acoustic Systems, Model Delta 143S, Austin, TX) for environment acclamations. The sound room was well ventilated and controlled with a light/dark cycle of 12:12 h. The temperature and humidity of the room were monitored daily. Each rat was placed in an individual wire cage (38 cm × 28 cm × 22 cm) that ensured acoustic transparency. Six cages at a time were placed next to each other in the sound room. At the 60 days of age when the tonotopic map in the primary auditory cortex has fully matured (Chang et al., 2005; Zhou et al., 2008), broad-band “white” noise (pseudo-random, 4–45 kHz in frequency range) was broadcasted continuously 24 h through two electrostatic speakers (ES1, Tucker-Davis Technologies (TDT), Alachua, FL) hanging 10 cm above the cages.

**Figure 1:**
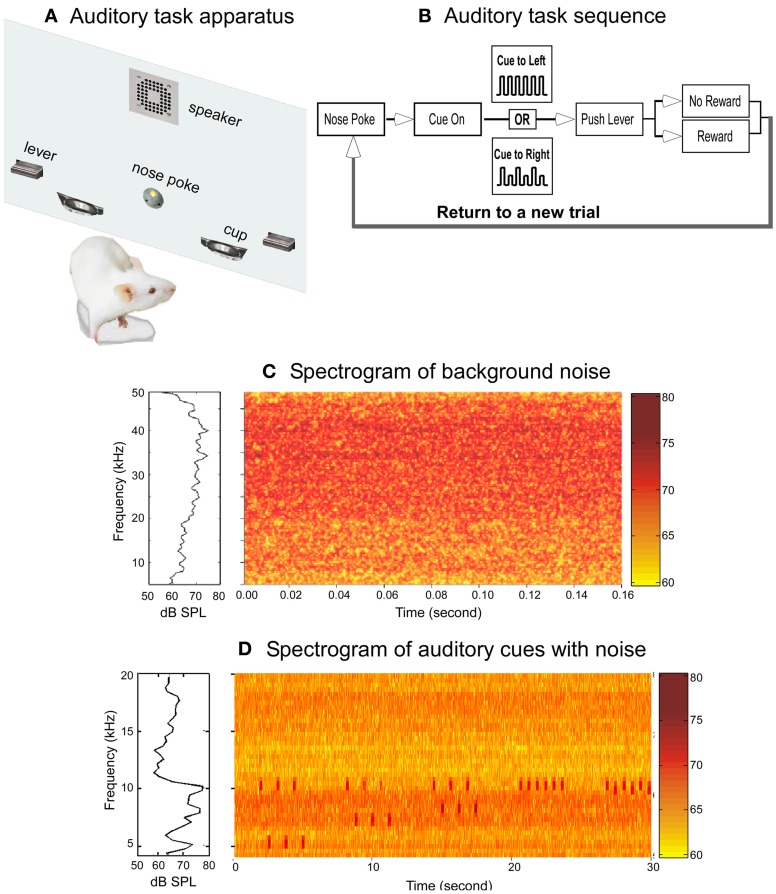
**The auditory behavioral task and the acoustic environments. (A)** Schematic drawing of the behavioral training chamber panel holding the operant devices. **(B)** Block diagram showing the auditory task sequence. **(C)** Spectrum (left panel) and spectrogram (right panel) of the noise to which the rats were exposed. The data represent the averaged measurements from the center of six cages. **(D)** Spectrum (left panel) and spectrogram (right panel) of the auditory cues and the noise background. To display the auditory cues clearly, only frequency range of 4–20 kHz was plotted.

The noise was generated by an Enhanced Real-Time Processor (RP2.1, TDT), attenuated with a Programmable Attenuator (PA5, TDT), and delivered to the speakers by an Electrostatic Speaker Driver (ED1, TDT). The noise level measured with a condenser microphone (ACO Pacific, Inc., Belmont, CA) at the level of rat (~5 cm off the cage floor) at the center of each cage ranged from 60 to 70 dB SPL (decibels sound pressure level, RMS) across frequencies from 4 to 45 kHz. The pattern of the noise measured for each cage was very similar and the averaged spectrogram across six cages is shown in Figure 1C.

### Electrophysiology

After 30 days of living in the noisy environment, standard *in vivo* electrophysiological methods were used to assess response properties of neurons in the primary auditory cortex. Rats were pre-treated with a mixture of Acepromazine (0.5 mg/kg), Xylazine (2 mg/kg) and Glycopyrolate (0.01 mg/kg) for tranquilizing, analgesia and bronchial secretion reduction. Rats were then anesthetized with 1.0–1.5% isoflurane in a mixture of oxygen/nitrous oxide (60:40%). The level of isoflurane was adjusted throughout the experiment to maintain the animal in an areflexic state. The head was fixed on a stereotaxic frame with a rat gas anesthesia head holder equipped with a palate tooth bar (David Kopf Instruments, Tujunga, CA) for securing the skull while leaving the ears open, allowing insertion of a plastic tube for sound stimulation. The skin on the top of the skull was incised to expose suture marks (center suture, Bregma, and Lambda) on the skull. The head was then leveled on the anterior-posterior and medial-lateral plane by using a rat alignment tool (Model 944, David Kopf Instruments, Tujunga, CA). The head was then turned 90° for surgical exposure of the auditory cortex during which the temporalis muscle was reflected, a craniotomy was opened over the auditory cortex, and the dura was removed. The exposed cortex was protected from desiccation with a thin layer of silicone oil. All recordings were conducted in a double-walled sound-proof room (IAC, Industrial Acoustics Company, Bronx, NY).

Single tungsten microelectrodes (0.5–1.5 MΩ, FHC) and/or sixteen-channel silicone probe (4 shanks by 4 sites, Model a 4×4–3 mm100–177, NeuroNexus Technologies, Ann Arbor, MI) were used for recording cortical neural responses to sound delivered via an ear tube inserted into the contralateral ear cannel. The other end of the ear tube was connected to a calibrated electrostatic speaker (EC1, TDT). The center of the primary auditory cortex was located by using the stereotaxic coordinates (AP ~ −5.0 mm and DV~ −4.0 mm; Paxinos and Watson, The Rat Brain, 4^th^ edition, Academic Press, 1998) and then confirmed with characteristics of stimulus-driven responses, such as response latency, the shape of turning curves, and response threshold (Doron et al., 2002). The electrode was lowered orthogonally into the brain with a micropositioner (Model 662, David Kopf Instruments, Tujunga, CA) and advanced in 10–50 μm steps until reaching cortical layer 4/5 (500–850 μm). Frequency tuning properties of well-isolated single neurons or multiple units with high signal/noise ratio (>3) were assessed and recorded with an automated computer program (OpenEx Suite, TDT), which generates sound stimuli and acquires neural responses. The neural signals were amplified (10,000X), filtered (0.3–6 kHz), and stored for off-line data analysis.

Frequency tuning properties of cortical neurons were characterized by measuring their responses to pure tone pips (50 ms in duration, 10 ms rise/fall time, 1.0–1.3 per sec repetition rate, 10 repetitions) with frequency range from 4 to 60 kHz (1 or 2 kHz step) and intensity level 0–80 dB SPL (10 dB step). To obtain the tonotopic map, electrodes were moved systematically across the primary auditory cortex with 100–200 μm steps between recording sites. Blood vessels were carefully avoided by adjusting the electrode placement step size.

The tone pips of each frequency/intensity pair were generated through the OpenEx Suite, calibrated automatically with a calibration file for each speaker (SigCal, TDT), and delivered pseudo-randomly through the ear tube. The sound calibration file was generated by measuring the output of the ear tube with a condenser microphone (ACO, Pacific, Inc., Belmont, CA) placed 1–2 mm away from the end of the tube. Calibration was conducted frequently to update the calibration file.

At the end of each recording session, single or multiple electrical lesions were made on the recording sites. Rat was perfused and the brain was extracted after three days of post-fixation with 4% of Formaldehyde solution. Standard Nissl staining protocol was used to visualize the lesion marks (Figure 4C).

### Auditory behavioral task

We developed a two-alternative, forced choice auditory operant task (Talwar and Gerstein, 1998, 1999), to examine the effects of noise-exposure on frequency discrimination (Zheng and Ycu, 2012). Rats were prepared for auditory task training by limiting food supplies until their body weight reached a stable level of 80–85% of the normal weight. After 30 days of living in the noisy environment, the rats were taken out for 30 min of daily training and returned to the noise room afterward.

The behavioral chamber (Figure 1A), located in a double-walled sound-proof room (IAC, Industrial Acoustics Company, Bronx, NY), was custom-made (Med-Associates Inc., St. Albans, VT) to be acoustically transparent. A calibrated speaker (FT17H, Fostex Corp., Norwalk, CA), mounted on the top-middle section of the operant panel, was used to broadcast auditory cues. A retractable lever (ENV-116RM, Med Associates Inc.) was mounted on each side of the panel. Pushing the lever triggers a dispenser (ENV-203-45IR, Med Associates Inc.) to delivery of a full nutritional pellet reward (45 mg Dustless Precision Pellets, Bio-Serv, Frenchtown, NJ) into a food cup located adjacent to each lever. *Ad libitum* water was supplied throughout each training session.

The auditory task is illustrated in Figure 1B. The rats were trained to poke their nose into a hole to start a trial. Nose poke triggers broadcasting of the auditory cues. Upon recognition of the cues, the rats need to approach one of the two levers, wait for the lever to extend into the chamber, and then push the lever within 2 s to obtain food rewards. Both levers emerged simultaneously at the offset of the auditory cues, but only one of the levers would trigger a food pellet if pressed. Auditory cues indicated which lever would produce a pellet. The auditory cue was one of two patterns of 3.2 s tone trains: one constant pattern consisted of six 10 kHz tones and the other alternating pattern in which the tones varied between 10 kHz and a lower frequency (5, 6, 7, or 8 kHz tones, corresponding to a frequency change (Δ*F*) of 50%, 40%, 30%, and 20%, respectively). Each tone pip, calibrated to be ~75 dB SPL, was 200 ms in duration and presented with a tone pip interval 400 ms. In each session, the two sound patterns were presented randomly with a probability of 0.5. A well trained rat would approach either the right or left lever based on the pattern of auditory cue to obtain food reward: a constant tone train for the left side and an alternating tone train for the right side (Figure 1B).

There were three possible outcomes in each trial: hit, miss, or false. In a hit trial, the rat pushed the lever indicated by the auditory cue within 2 s after the lever emerged. In a false trial, the rat pushed the lever on the side opposite that indicated by the cue. In a miss trial, the rat did not push either lever within 2 s after the lever emerged. Lever push, either hit or false, or expiration of the 2 s time window in a miss trial, resulted in retraction of both levers, signaling the end of a trial. The rat would initiate a new trial by poking its nose into the nose poke hole. Since each trial was initiated by the rat, a trained rat would always pursue pushing a lever, resulting in no missing trials in almost all the sessions. The performance of a rat was quantified by calculating the hit rate for each sound pattern in each training session:

Hit Rate = (Number of hits for each sound pattern/Total number of trials for each sound pattern) × 100.

The behavioral training and testing were fully automated through the Med PC program (Med-Associates Inc.). Each event (nose-poke in/out, cue on/off, lever in/out, lever push) during behavioral task was time stamped with a 10 ms resolution and recorded for off-line analysis. Each training and testing session was closely monitored via a SONY monitor placed outside of the sound room.

Based on our early observations in behavioral training of control rats that most rats reached a stable performance level around 75% hitting rate and previous pitch discrimination studies on rats by others indicating a plateau performance around 75% of success rate (Sloan et al., 2009), a rat was judged as having learned the task when the performance was stable at a 75% hitting rate for at least three consecutive training sessions.

### Fine frequency discrimination tests

After having learned the auditory task with stable performance of discriminating the large Δ*F*s (20–50%), the rats were tested with smaller frequency variations (Δ*F* = 3%, 5%, 8%, and 10%). In a test session, only one of the alternating tone trains with smaller Δ*F* was presented in 10% of the total trials, randomly interleaved with other tone trains of constant frequency or large Δ*F*s. The hit rate for each testing sound pattern was calculated after each testing session.

### Testing in a noisy environment

To examine the performance of the rats in a noisy environment, background noise (broad-band, pseudo-random noise, 4–45 kHz in frequency range) was added to the sound room by an electrostatic speaker (ES1, TDT) hanging above the behavioral chamber. The noise was generated by an Enhanced Real-Time Processor (RP2.1, TDT), attenuated with a Programmable Attenuator (PA5, TDT), and delivered to the speaker via an Electrostatic Speaker Driver (ED1, TDT). The noise level measured at the level of the rat height was 60–70 dB SPL (decibels in sound pressure level, RMS), giving an averaged auditory cue-to-background noise ratio of approximately 1.2 (Figure 1D).

### Data analysis

Because many studies have demonstrated auditory task training induced plasticity in the primary auditory cortex (Edeline and Weinberger, 1993; Edeline et al., 1993; Bakin et al., 1996; Blake et al., 2002, 2006; Beitel et al., 2003; Bao et al., 2004; Fritz et al., 2005, 2007; Polley et al., 2006; Dahmen and King, 2007; Van Wassenhove and Nagarajan, 2007; Weinberger, 2007; Zhou and Merzenich, 2007, 2009; Berlau and Weinberger, 2008; De Villers-Sidani et al., 2010), in order to examine the effect of noisy exposure on the tonotopic map, the electrophysiological data reported here were obtained from the untrained rats (14 unexposed rats and 10 noise-exposed rats). In two noise-exposed rats, response properties of A1 neurons were examined after behavioral training and testing. We did not observe any training effects on the neuronal response properties in these two rats, possibly due to returning to the noisy room after daily 30 min training throughout the experiment. Thus, the electrophysiological data from these two rats were pooled into the data obtained from the untrained, noise-exposed group, giving a total of 12 rats in the noise-exposed group for electrophysiology data.

Recorded electrophysiology and behavioral data were extracted and analyzed using MatLab (MathWorks, Inc., Natick, MA). Statistical analyses were conducted using Sigma Plot and Sigma Stat (Systat Software, Inc., Chicago, IL). OpenSorter (TDT) was used for off-line spike sorting from multi-unit recordings. No difference was found between single and multi-unit recordings on the response properties analyzed and data were thus pooled together.

The best frequency of each recording site was defined as the tone frequency at which reliable responses were elicited with lowest tone level. The lowest tone level was taken as the response threshold. For sites with two frequency response regions at the bottom tip of the receptive field, the mean value of these two frequencies was taken as the best frequency. For sites with more than two frequency response regions at the bottom tip of the receptive field, the median value of these frequencies was taken as the best frequency. For a few sites exhibiting double peak tuning curves, the best frequencies were taken from the peak with lower threshold.

To construct the tonotopic map, the distance between the most rostral-caudal and dorsal-central recording sites was normalized and the best frequencies were plotted as a function of the normalized distance. Unresponsive sites to tonal stimulation were used as reference for the boundary of A1 but not plotted in the tonotopic map. There was slight variation (<2°) in the rostral-caudal axis direction among different rats, but was not corrected for constructing the tonotopic map. The correlation coefficient between the best frequency and the rostral-caudal axis of the A1 was also calculated for each rat.

To examine whether the best frequencies in the A1 of noise-exposed rats is represented randomly, we calculated the Euclidean distance matrix (Matlab function pdist) for each recorded site in each noise-exposed animal with three parameters: the spatial location coordinates (along the dorsal-ventral and caudal-rostral direction) in the A1 and the best frequency. We then randomized the assignments of the best frequencies to the spatial location coordinates within each rat 100 times and re-calculated the Euclidean distance matrix for each site in each randomized data set. The values of the first six nearest distance for the sites with the same best frequency were then compared between the experimental data and the randomized data.

The behavioral patterns of rats performing the auditory task were quantitatively characterized with two measurements: reaction time and temporal variation of performance within a session. The reaction time, reflecting mainly an animal's attentiveness to the task, was calculated as time elapsed between the offset of auditory cue and the lever push. The temporal variation of performance within a session was analyzed by counting the accumulative number of hits at three different stages (10 min per stage) of a single session (30 min): early, middle, and late. This measurement represents the dynamic patterns of an animal's overall performance and reflects improvement/adaptation that might have occurred within a single session. Additionally, the total number of trials executed in a single session, reflecting several aspects of an animal's state, including motivation, concentration, and persistence, was also analyzed to assess animal's overall performance in different testing conditions.

## Results

Rats were placed in a sound-proof room at the age of 60 days when their auditory system is fully maturated (Chang et al., 2005; De Villers-Sidani et al., 2007). Rats develop quickly after birth and reach sexual maturity at about 6–7 weeks old. Electrophysiology studies have shown that the response properties of neurons in A1, such as sharpness of frequency tuning curve, threshold, response latency, and size of A1, reached stable level at the age of postnatal 15 days (De Villers-Sidani et al., 2007). To study the effects of chronic noise-exposure in adult rats, we have thus used 60 days old rats and referred them as adults.

Each rat was housed in an individual wire cage adjacent to the other cages. Continuous, broad-band white noise (pseudo-random, 4–45 kHz; Figure 1C) was broadcast through two speakers mounted above the wire cages. This low-level noise (60–70 dB SPL) would cause minimal physical damages to the rat's inner ear while masking the rat's vocal communications (Takahashi et al., 2010).

### Reorganization of the tonotopic map in the noise-exposed rats

**Figure 2:**
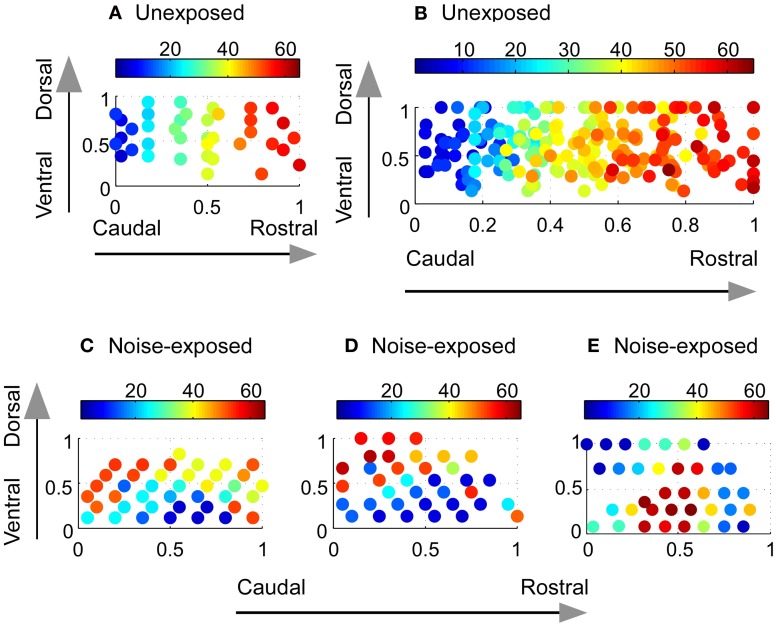
**Reorganization of the tonotopic map in the A1 of adult rats chronically exposed to low-level noise. (A–B)** Tonotopic map in the A1 of an unexposed rat **(A)** and compound frequency representation in the A1 of all unexposed rats **(B)**. **(C–E)** Examples of reorganized frequency representation in three noise-exposed rats. The color bar indicates the value of sound frequencies. The x- and y-axes represent normalized caudal—rostral and ventral—dorsal dimension, respectively, of the primary auditory cortex (see “Materials and Methods”).

**Figure 3:**
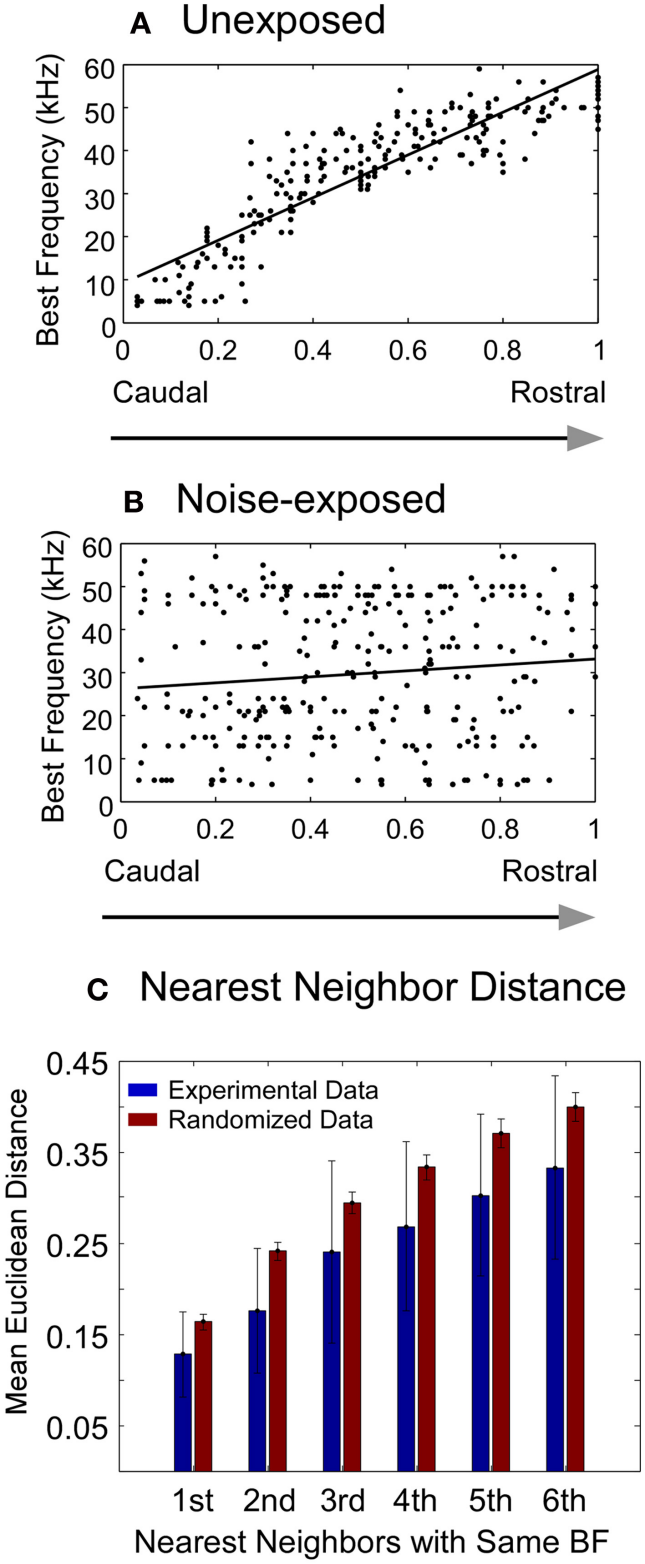
**Changes in best frequency representation in the A1 of adult rats chronically exposed to low-level noise. (A–B)** Best frequency representation along the caudal-rostral axis of the A1 in unexposed **(A)** and noise-exposed **(B)** rats. The x- and y- axes represent normalized caudal—rostral and ventral—dorsal dimension, respectively, of the primary auditory cortex (see “Materials and Methods”). **(C)** Comparison of the mean Euclidean distance of the recorded sites with the same best frequency between the experimental data from the noise-exposed rats (blue bars) and the randomized data (red bars) (see “Materials and Methods”). Vertical bar: mean ±1 SD. All the pairs of bars are significantly different from each other (Mann–Whitney Rank Sum Test, *P* < 0.001).

To investigate the effect of noise exposure on the tonotopic map, we conducted *in vivo* electrophysiological recording in the primary auditory cortex (A1) in the rats after 30 days of noise exposure (see “Materials and Methods”). A tonotopic map from an untrained, unexposed rat is shown in Figure 2A. As the recording electrode was moved along the caudal → rostral axis, the best frequencies of the recording sites also changed systematically from low to high frequency range. Recordings along the dorsal-ventral axis revealed receptive fields with similar best frequency values. Thus, consistent with previous studies (Doron et al., 2002), a systematic progression of frequency representation along the caudal–rostral axis and the iso-frequency contours along the ventral-dorsal axis of the A1 were evident. Figure 2B illustrates the compound tonotopic map obtained from all the untrained, unexposed rats (*n* = 14). The highly correlated distribution of the best frequencies along the caudal-rostral axis of the A1 in all the untrained, unexposed rats is plotted in Figure 3A (Spearman's rank order correlation test: correlation coefficient = 0.902, *p* = 0.0000002, *n* = 242).

**Figure 4:**
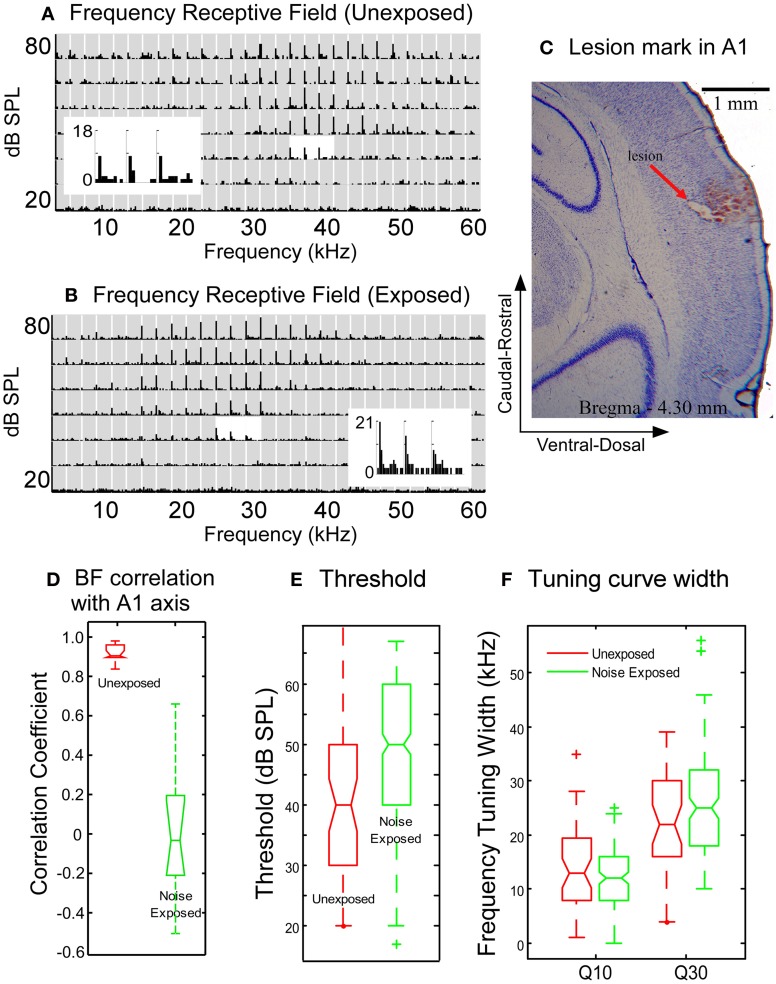
**Comparison of neuronal response properties between the untrained, unexposed and the noise-exposed rats. (A–B)** Examples of frequency receptive fields recorded from the A1 of an unexposed **(A)** and a noise-exposed rat **(B)**. ***Gray rectangles*** in the plots of receptive field: post-stimulus histogram (PSTH) of neuronal responses to each combination of frequency/intensity (bin with: 10 ms, duration: 750 ms). The PSTHs around the best frequency at the threshold level were enlarged to show the details of neuronal responses. **(C)** Nissl stain showing the lesion mark of recording site in a noise-exposed rat. **(D)** Box plot comparison of the correlation coefficients of best frequency distribution along the caudal-rostral axis of the A1. **(E)** The tone response threshold in noise-exposed rats increased moderately as compared with the unexposed rats. **(F)** The width of frequency tuning curves measured at Q10 and Q30 in the noise-exposed rats remained the same as that in the untrained, unexposed rats. In the box plot in **D–F**, the upper and lower lines of the notched box represent the 25^th^ and 75^th^ percentiles of the data and the middle line indicates the median. The horizontal lines above and below the notched box represent the range of the data sample. Outliers, defined as more than 1.5 times the inter-quartile range (the length of the box) away from the top or bottom of the box, were plotted with symbols “+”. No-overlapping notches between the two data sets indicate significant difference in medians with 95% confidence.

In contrast, the tonotopic map of the noise-exposed rats was dramatically reorganized. Three examples of the reorganized map are illustrated in Figures 2C–E. There were no systematical changes in the best frequencies of the receptive fields along the caudal → rostral direction. The best frequencies also changed along the dorsal-ventral axis. Consequently, the tonotopic map seen in the untrained, unexposed rats was replaced with an apparently random, but spatially clustered, representation of the best frequencies in the A1 of noise-exposed rats. The high correlation of the best frequency with the caudal-rostral axis of the A1 in the untrained, unexposed rats was consequently absent in noise-exposed rats (Figure 3B, Spearman's rank order correlation test: correlation coefficient = 0.095, *p* = 0.00946, *n* = 307). The population distribution of the correlation coefficients between the best frequency and the caudal-rostral axis of the A1 for the untrained, unexposed and the noise-exposed rats is compared with a box plot in Figure 4D.

To examine whether the best frequencies in the A1 of noise-exposed rats was represented randomly or spatially clustered, we calculated the Euclidean distance matrix for each recorded site in each noise-exposed animal. The mean Euclidean distance of the first six nearest recorded sites with the same best frequency for all the noise-exposed rats was compared with that from a randomized data set (see “Materials and Methods”). As shown in Figure 3C, the mean Euclidean distance from the experimental data was significantly smaller than that from the randomized data (Mann–Whitney Rank Sum Test, *p* < 0.001 for all six pairs). Thus, the best frequencies in the A1 of noise-exposed rats were not represented randomly. As exemplified by the individual cases in Figures 2C–E, the best frequencies were apparently represented in a spatially patched/clustered fashion.

The response threshold of neurons in the A1 of the noise-exposed rats was, on average, significantly higher than that in the untrained, unexposed rats (Figure 4E, 50.9 ± 12.3 vs. 40.2 ± 13.7, Mann–Whitney Rank Sum Test, *p* = <0.001). The width of frequency tuning in the A1 of the noise-exposed rats (Figure 4B) was, however, not significantly different from that in the untrained, unexposed rats (Figure 4A) at both sound levels of 10 dB (Q10) and 30 dB (Q30) above threshold (Figure 4F; exposed vs. unexposed, at Q10:12.6 ± 5.1 vs. 13.4 ± 7.7, *p* = 0.6899; at Q30:26.3 ± 10.4 vs. 23.2 ± 9.4, *p* = 0.2185; Mann–Whitney Rank Sum Test). These results indicate that the reorganization of the A1 was not caused by broadening of frequency tuning. Instead, frequency tuning properties, particularly the sharpness of frequency tuning curves, remained largely unchanged while the tonotopic map underwent dramatic reorganization.

### Normal auditory task learning in the noise-exposed rats

To investigate the effects of chronic noise-exposure on frequency discrimination, we trained rats in a two-alternative forced choice operant auditory task (Figures 1A and B) modified from a previous study (Talwar and Gerstein, 1998). Nine unexposed (referred to as “*control*” thereafter) and six noise-exposed rats were trained to poke their nose into a hole to start a trial, discriminate two sound patterns, and then push a lever indicated by the auditory cue to obtain a food reward (see “Materials and Methods” for details).

**Figure 5:**
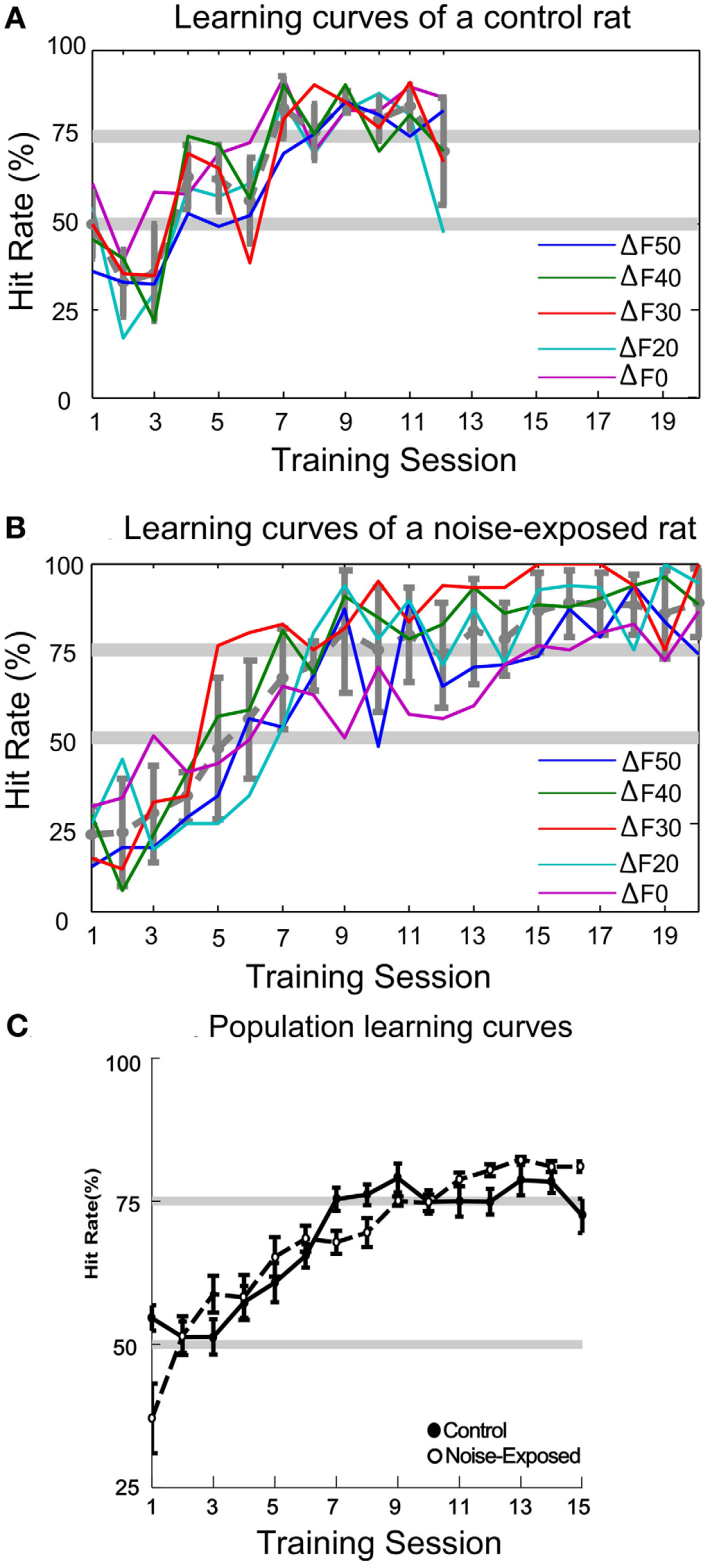
**Auditory task learning curves. (A–B)** Learning curves for a control and a noise-exposed rat, respectively. Each colored line represents the learning progress for recognizing each sound pattern, indicated by the percent frequency change (Δ*F* = (low – high)/high frequency; Δ*F*0 represents the constant sound pattern). Both control and noise-exposed rats learned to recognize all patterns of tone trains concurrently. The average hit rate across all sound patterns in each session is shown by the dashed-gray line (error bars: mean ±1SD). **(C)** Population learning curves of control and noise-exposed rats. No significant difference in hit rate was found in all sessions between the control and noise-exposed rats (error bars: mean ±1 SEM).

Typical learning curves of a control and a noise-exposed rat are illustrated in Figures 5A and 5B, respectively. Each colored thin line depicts the learning progression for different sound patterns, as indicated by the value of Δ*F*. It is clear that, for both the control and the noise-exposed rat, the learning progression is concurrent across all sound patterns. The thick gray line depicts the averaged learning progress across all sound patterns. The averaged population learning curves for control (*n* = 9) and noise-exposed (*n* = 6) rats are plotted in Figure 5C. On average, it took about seven sessions of 30 min training for the control rats to reach the 75% correct criterion (see “Materials and Methods”). The noise-exposed rats showed a similar learning pattern to the control rats, reaching the 75% criterion at sessions nine. Statistically, however, the hitting rates between the two groups of rats are not different at all training sessions (Mann–Whitney Rank Sum Test, *p* > 0.05). Thus, both groups of rats learned the task with similar speed and patterns.

### Impaired fine pitch discrimination in the noise-exposed rats

**Figure 6:**
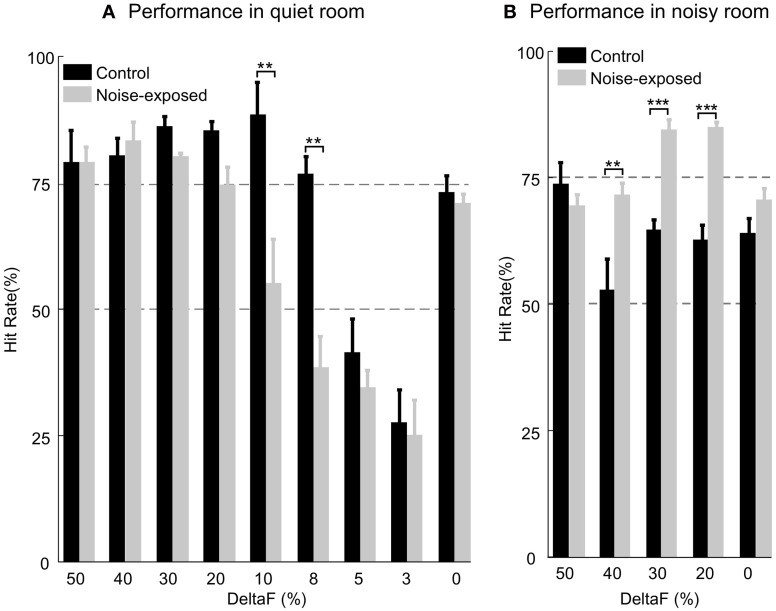
**Comparison of auditory task performance in a quiet (A) and a noisy room (B).** In a quiet room, the noise-exposed performed significantly worse than the control rats only at the Δ*F* = 10% and 8%. In a noisy room, the noise-exposed rats performed significantly better than the control rats at the most of Δ*F*s (40%, 30%, and 20%) tested. Error bars: mean ±1 SEM. Number of asterisks indicates the level of significance of Mann–Whitney Rank Sum Test: ^**^, *P* < 0.01; ^***^, *P* < 0.001.

After the rats had learnt the task with stable performance, we tested their ability of discriminating fine frequency variations by presenting alternating tone trains with smaller frequency variations (Δ*F* = 10%, 8%, 5%, and 3%). In a test session, only one of these alternating tone trains was presented randomly-interleaved with other tone trains that the rat had been trained with (Δ*F* = 0%, and 20–50% in 10% steps). Six noise-exposed and three control rats were tested with different number of sessions for each small Δ*F*. On average, each small Δ*F* was tested in seven sessions for both groups of rats. As shown in Figure 6A, the noise-exposed rats performed equally well as the control rats in detecting large frequency changes (Δ*F*s = 20–50%) but performed significantly worse than the control rats in detecting small frequency changes (Δ*F*s = 10% and 8%). At these small Δ*F*s, while the control rats performed above the 75% hit rate, the performance of the noise-exposed rats had dropped close to or below the chance level (Mann–Whitney Rank Sum Test, *p* = 0.002 and 0.007 at Δ*F* = 10% and 8%, respectively, between the control and noise-exposed rats). When tested with Δ*F*s = 5% and 3%, values around/below the frequency discrimination threshold reported from other studies in rats (Syka et al., 1996; Talwar and Gerstein, 1998; Sloan et al., 2009), both groups of rats failed to discriminate the sound patterns. Thus, the noise-exposed rats exhibited impairment in fine, but not coarse, pitch discrimination as compared to the control rats.

### Consistent performance of the noise-exposed rats in a noisy environment

We tested the performance of a subset of the control (*n* = 3; 7 testing sessions for each rat and total 21 sessions for all) and all of the trained, noise-exposed rats (*n* = 6; 7–13 testing sessions for each rat and total 60 sessions for all) in an environment filled with low-level noise (see “Materials and Methods”). In these tests, the Δ*F*s (50%, 40%, 30%, and 20%) at which the two groups of rats performed equally well in a quiet room (Figure 6A and Table 1) were used. In a noisy environment, the noise-exposed rats performed significantly better than the control rats did for most of the sound patterns tested (Figure 6B and Table 1; *p* = 0.008 for Δ*F* = 40% and *p* < 0.001 for Δ*F* = 30% and 20%; Mann–Whitney Rank Sum Test). For the noise-exposed rats, there was no significant difference at each of the tested Δ*F*s between the two testing conditions (Table 1). In contrast, for the control rats, the difference in hit rate between the two testing conditions was significant at most of the tested Δ*F*s (Table 1). Thus, while the control rats performed poorly in a noisy environment, the noise-exposed rats performed consistently in both a quiet and a noisy environment.

**Table 1:** **Comparison of performance between the control and noise-exposed rats in a quiet and a noisy environment**.

**Δ***F***s**		**50%**	**40%**	**30%**	**20%**	**Grand Mean**
Control (*N* = 9)	Quiet (*n* = 9)	78.3 ± 2.4	80.0 ± 2.2	86.1 ± 1.3	85.0 ± 4.4	81.3 ± 1.5
			^***^	^***^	^***^	
	Noisy (*n* = 3)	73.6 ± 8.2	52.8 ± 14.5	64.8 ± 5.6	62.7 ± 7.2	62.4 ± 9.4
			^**^	^***^	^***^	
Noise-exposed (*N* = 6)	Noisy (*n* = 6)	69.5 ± 3.5	72.0 ± 4.8	84.2 ± 4.1	84.9 ± 2.6	77.1 ± 4.9
	Quiet (*n* = 6)	78.8 ± 6.5	83.1 ± 3.8	80.1 ± 0.7	74.4 ± 3.2	79.7 ± 4.4

The letter n indicates that n out of N rats were tested in each condition (quiet or noisy environment). Each value (mean ± SEM) is the averaged hit rate across the tested rats for each ΔF. The asterisks indicate statistically significant difference between the two adjacent groups: [(control || quiet) vs. (control || noisy)] and [(control || noisy) vs. (noise-exposed || noisy)]. Number of asterisks indicates the level of significance of Mann–Whitney Rank Sum Test:

** , P < 0.01;

*** , P < 0.001. The grand mean is the averaged hit rate across all the tested ΔFs for each experimental group (across row).

### Similar behavioral patterns of the control rats in a quiet and a noisy environment

**Figure 7:**
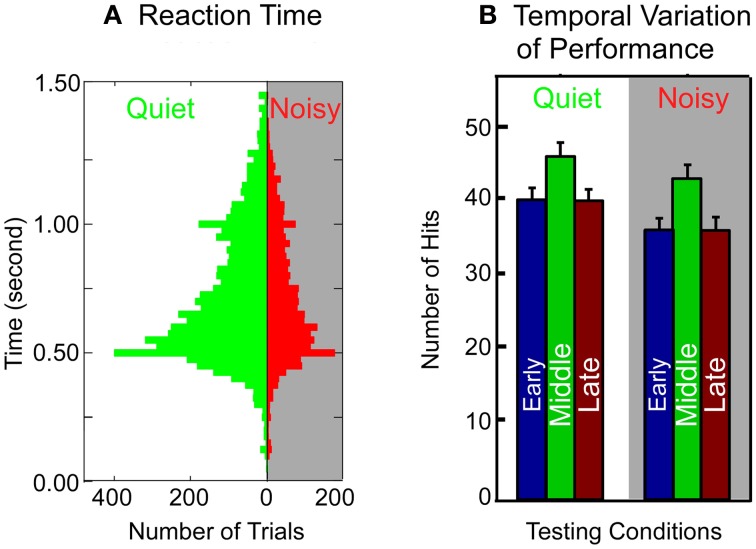
**Comparison of behavioral patterns of the control rats in a quiet and a noisy environment. (A)** Reaction time. The reaction time is measured as the time elapsed between the offset of the auditory cue and lever push. Histogram bin width: 100 ms. **(B)** Temporal patterns of performance within a session. Each 30 min session is divided into three 10 min stages (early, middle, and late). The accumulative number of hits is averaged across all the control rats through multiple sessions. Error bars: mean ±1 SD.

To determine whether stress or distraction may have caused the poor performance of the control rats in the noisy testing environment, we analyzed the animal's behavioral patterns in two testing conditions by measuring reaction time and temporal variation of performance within a session (see “Materials and Methods”). If the control rats had been stressed or distracted by background noise, a significant difference in the above measurements between the two testing conditions (quiet vs. noisy) would have been evident. However, as shown in Figure 7, no significant difference was seen in the behavioral patterns between the two testing conditions. The overall distribution of reaction time (Figure 7A) from all control rats tested in a noisy environment was very similar to that in a quiet environment (mean 0.4945 ± 0.2028 vs. 0.5055 ± 0.2152 s; *p* = 0.3864, Mann–Whitney Rank Sum Test). The pattern of temporal variation in performance, indicated by the accumulated number of hits in early, middle, and late stage of each 30 min testing session (Figure 7B), was also similar between the two testing conditions for the control rats. The total number of hits in a noisy environment was, however, significantly less than that in a quiet room (noisy vs. quiet: 116 ± 18 vs. 130 ± 24; *p* = 0.023, Mann–Whitney Rank Sum Test). In addition, the total number of trials per session was not significantly different between the two testing conditions for the control rats (quiet vs. noisy: 180 ± 20 SD vs. 174 ± 31 SD; *p* = 0.9886, Mann–Whitney Rank Sum Test). Thus, the decreased total number of hits in the noisy environment was not due to a reduced number of trials executed by the control rats but, instead, due to failure in frequency discrimination. Together, the results of these behavioral analyses argue strongly that the deteriorated performance of the control rats in a noisy environment was unlikely caused by stress or distraction.

## Discussion

The results of this study demonstrate that exposure to low-level ambient noises induced a dramatic reorganization of the tonotopic map in the primary auditory cortex of adult rats. Similar large-scale reorganization of neural networks in the A1 induced by sound exposure has also been reported recently in adult cats (Pienkowski and Eggermont, 2009, 2010a,c). Collectively, these lines of studies demonstrate that brain in adult animals maintains a large degree of plasticity. We further examined the perceptual consequence of adult brain plasticity. The behavioral testing results demonstrate that exposure to low-level ambient noises to adult rats impaired fine pitch discrimination, suggesting that the tonotopic map might be necessary for fine pitch discrimination. On the other hand, the ability to discriminate large pitch variations without the benefit of a tonotopic map in the noise-exposed rats suggests that cortical map might not be necessary for coarse discrimination of sensory information. Moreover, by testing the animals' performance in a noisy environment, the results revealed a possible adaptive link between brain plasticity and perception.

Sensory experience is most effective in shaping the brain function during the critical period. In the rat auditory system, a series of studies have shown that the effects of passive exposure of different patterns of sounds to rat pups depended not only on the time windows of developmental stages but also on the features of the sound, leading to the discovery of multiple-sensitive periods in the early development of the rat auditory system (Zhang et al., 2001, 2002; De Villers-Sidani et al., 2007; Insanally et al., 2009, 2010). Together, these studies have led to the prevailing view that the extent to which experience shapes brain function in adult animals is largely limited. To our best knowledge, extensive studies have, however, not been carried out to test this view. In most of the studies exposing sounds to rat pups, sound exposures were either restricted to a short period of time or stopped before the rats reached the age of 60 days (Chang and Merzenich, 2003). A series of recent studies (Pienkowski and Eggermont, 2009, 2010a,c) have, however, revealed a large degree of plasticity in the auditory cortex of adult cats when exposed to moderate-level tone pip ensembles for 7–13 weeks. The current study was designed to test whether similar degrees of plasticity can be seen in adult rats. Indeed, the results demonstrate clearly that passive sound exposure can induce a substantial plasticity in the primary auditory cortex of adult rats. A large body of studies has shown that the neural circuits in adult brain can be modified by behavioral training (Edeline and Weinberger, 1993; Edeline et al., 1993; Bakin et al., 1996; Blake et al., 2002, 2006; Beitel et al., 2003; Bao et al., 2004; Fritz et al., 2005, 2007; Polley et al., 2006; Dahmen and King, 2007; Van Wassenhove and Nagarajan, 2007; Weinberger, 2007; Zhou and Merzenich, 2007, 2009; Berlau and Weinberger, 2008; De Villers-Sidani et al., 2010). Intentional effort related to the reward systems has been considered as the primary driving force for the modification of the brain circuits (Keuroghlian and Knudsen, 2007). Interestingly, activation of the reward systems, i.e., dopaminergic (Bao et al., 2001) or cholinergic (Kilgard and Merzenich, 1998) pathways, by electrical stimulation combined with passive sound exposure was sufficient to induce plasticity in adult auditory cortex. As discussed later, it is possible that the reorganization of the tonotopic map in the noise-exposed adult rats could have been driven by activation of the reward systems through intentional efforts of the rats for vocal communications.

The noise-exposure induced dramatic reorganization of the A1 tonotopic map in the adult rats is different in nature from that in the rats raised with noisy environment (Chang and Merzenich, 2003). In the adult rats exposed to noise, the frequency tuning curve width remained unchanged while the tonotopic map underwent reorganization; in the rats raised with noise, the frequency tuning curve width was much broader than normally raised rats while the tonotopic map was prevented from development. Secondly, tonotopic representation of the best frequencies in the noise-exposed adult rats was apparently transformed into patches without tonotopy whereas in the noise-reared rats the tonotopic representation was never formed and the best frequencies were represented randomly. These fundamental differences indicate that the mechanisms underlying adult plasticity are different from those of the plasticity in the rats raised with noise. It would be interesting to examine whether the reorganized auditory map in adult rats can be recovered after removing the noise, like what has been shown in the rats raised with noise (Bao et al., 2003).

The finding that noise-exposed rats can detect large frequency changes embedded in a sequence of tone patterns suggests that the tonotopic organization in the primary auditory cortex is not necessary for perception of large frequency variations. Lesion studies have shown that the auditory cortex is not required for sound frequency discrimination (Ohl et al., 1999; Sutter and Shamma, 2011). It is therefore tenable that the reorganization in the primary auditory cortex in noise-exposed rats represents the primary level at which the brain modifies its functions induced by ambient noises. In contrast to the lesion studies, however, the noise-exposed rats also exhibited impairments in fine pitch discrimination, suggesting that a tonotopic map in A1 may be necessary at least for discriminating small scale pitch changes.

The poor performance of the control rats in a noisy environment can be caused by distraction and/or stress imposed by noisy background; the consistent performance of a noise-exposed rat could simply be due to the animal's acclimation to the noises. Detailed analysis of the patterns of behaviors did not, however, reveal difference between the testing conditions in the control rats (Figure 7). Therefore, it is unlikely that stress and/or distraction were major contributors to the poor performance of the control rats in a noisy environment. Together with the finding that the noise-exposed rats exhibited a consistent level of performance in both quiet and noisy conditions, it is strongly suggestive that the reorganized auditory map—and perhaps, the whole central auditory system (see below)—might have served as the neural substrate for the consistent performance of the noise-exposed rats in a noisy environment. Map reorganization revealed in this study may therefore have an adaptive role in nature; a surmise that warrants future systematic investigation. It is, however, important to emphasize that the results of current studies do not prove the causality between the map reorganization in A1 and behavioral outcomes in both quiet and noisy environment. Further systematic studies, such as pinpointing the locus/loci of the plasticity induced by noise-exposure, testing behavioral performance after restoring the tonotopic map in A1—possibly by removing the animals from noisy room, behavioral training, enriching acoustic experience in a quiet environment, or combination of all these means—are needed to establish the causality link between the brain plasticity and behavioral outcomes.

The neural mechanisms underlying the large-scale reorganization of the tonotopic map in adult rats exposed to low-level noise is currently unknown; but one plausible mechanism might be plasticity of the inhibitory networks. Inhibition not only shapes response properties of cortical neurons (Metherate and Ashe, 1995; Horikawa et al., 1996; Tan et al., 2004; Wehr and Zador, 2005; Kurt et al., 2006; Caspary et al., 2008; Moeller et al., 2010; Razak and Fuzessery, 2010; Sadagopan and Wang, 2010; Ye et al., 2010; Zhou et al., 2010), but also plays an important role in experience-dependent plasticity (Zheng and Knudsen, 1999, 2001; Luscher et al., 2011). Cortical neurons in the primary auditory cortex receive a large number of excitatory inputs. Inhibitory networks sculpt the broad excitatory connections to give rise to the sharp representation of sound frequencies (Tan et al., 2004). It is thus possible that changes in the pattern of inhibitory networks alone might be sufficient to reorganize the tonotopic map in noise-exposed adult rats. Equally plausible is remodeling of the excitatory connections in the A1 that gives rise to the large-scale reorganization of tonotopic map. Many studies have demonstrated the structural remodeling of neural circuits induced by experience through a variety of mechanisms (Barnes and Finnerty, 2010) such as axonal growth (Debello et al., 2001), synapse genesis (Foscarin et al., 2011), change in firing property (Miller et al., 2011), and modifications in neurotransmitter and/or neuromodulator systems (Rebola et al., 2010; Kazlauckas et al., 2011; Salgado et al., 2011).

The reorganization of the tonotopic map in noise-expose adult rats might also be a reflection of subcortical changes along the central auditory pathways. Functional and structural plasticity has been reported in almost all subcortical auditory structures (Echteler et al., 1989; Edeline and Weinberger, 1991; Edeline, 1999; Barsz et al., 2007; Speechley et al., 2007; Werthat et al., 2008). In fact, our previous studies of c-fos immunocyctochemistry staining (Zheng, 2004) showed that, although the tonotopic representation is still intact, the bandwidth of frequency representation in dorsal cochlear nucleus was broadened in noise-exposed rats. These lines of evidence support the notion that low-level noise-exposure also induces plasticity in subcortical auditory structures. Integration of these changes along the central auditory pathway would have resulted in the large-scale map reorganization in the A1 of noise-exposed rats.

The driving force for reorganizing the tonotopic map is likely to be the random bombardments by the constant random noisy inputs to the networks of the central auditory system. It has been shown that exposure to temporally-patterned noise pulses induces and instructs plasticity in temporal responses properties of auditory cortical neurons (Kilgard et al., 2001; Zhang et al., 2002; Insanally et al., 2009, 2010). Lack of regularity of the reorganized cluster representation of frequencies across individual noise-exposed rats is consistent with the notion that the pattern of reorganization in the primary auditory cortex is driven by the random noise inputs across frequency channels. Alternatively, it is also possible that the driving force behind the large-scale reorganization observed in this study is the increased effort for vocal communications. Since rats were hosted in wired-cages that are placed adjacent to each other, the rats could have engaged in vocal communication throughout the period of noise exposure, even though the vocalizations were not monitored. The masking effect of the noise over vocalizations might have forced the rats to increase their effort in order to recognize the vocalizations, which, in turn, served as a driving force to modify the networks of the central auditory system in a way to optimize detection of salient acoustic signals in a noisy environment. Intentional effort has been demonstrated to be a critical driving force for adult brain plasticity after the closure of the sensitive period (Bergan et al., 2005; Keuroghlian and Knudsen, 2007). It is thus plausible that the intentional efforts of vocal communications over the low-level ambient noises may have driven the large-scale reorganization in the central auditory system observed in noise-exposed adult rats.

An intriguing question is how a rat with a reorganized tonotopic map in A1 can even discriminate the large scale pitch changes. The simplest answer to this question could be that the A1 is not involved in discriminating large pitch changes as shown in lesion studies (Ohl et al., 1999; Sutter and Shamma, 2011); instead, large scale pitch discrimination was accomplished in subcortical nucleus/nuclei where the tonotopic organization remained unchanged in the noise exposed rats. An alternative hypothesis is that the reorganization of the A1 auditory map is in fact a manifestation of adaptation for extracting salient auditory information in a noisy environment. The patched best frequency representation in the A1 of noise-exposed rats (Figures 2C–E) resembles the “salt-pepper” representation of the preferred orientation in the primary visual cortex (V1) of rodents (Drager, 1975; Girman et al., 1999; Bonin et al., 2011; Tan et al., 2011), as contrast to the topographic orientation map in the V1 of primates (Ferster and Miller, 2000). A recent study in visual system (Hansel and Van Vreeswijk, 2012) suggested that the balanced excitatory and inhibitory inputs into neurons in the V1 of rodents can be sufficient for generating the sharp orientation selectivity without a functional orientation map. Continuous noise exposure might have driven the A1 network to re-establish a new balanced state of excitation and inhibition, which not only has given rise to the sharp frequency selectivity but also the patched representation of the best frequencies. Combination of the patched representation and the sharp frequency tuning could have provided the neural substrate for detecting pitch variations in a noisy environment. The reorganized tonotopic map may thus have served as the neural substrate for the significantly better performance of the noise-exposed rats than the control rats in a noisy environment, a speculation worthy of further investigations both experimentally and theoretically with computational models.

### Conflict of interest statement

The author declares that the research was conducted in the absence of any commercial or financial relationships that could be construed as a potential conflict of interest.
